# Effects of single-session perturbation-based balance training with progressive intensities on resilience and dynamic gait stability in healthy older adults

**DOI:** 10.3389/fbioe.2025.1642158

**Published:** 2025-08-29

**Authors:** Ringo Tang-Long Zhu, Friederike A. Schulte, Navrag B. Singh, Christina Zong-Hao Ma, Chris Awai Easthope, Deepak K. Ravi

**Affiliations:** ^1^ Department of Biomedical Engineering, The Hong Kong Polytechnic University, Kowloon, Hong Kong SAR, China; ^2^ Research Institute for Smart Ageing, The Hong Kong Polytechnic University, Kowloon, Hong Kong SAR, China; ^3^ Department of Rehabilitation, Southwest Hospital, Third Military Medical University (Army Medical University), Chongqing, China; ^4^ SturzZentrum Schweiz, Zurich, Switzerland; ^5^ Singapore-ETH Centre, Future Health Technologies Program, Singapore, Singapore; ^6^ Data Analytics & Rehabilitation Technology (DART), Lake Lucerne Institute, Vitznau, Switzerland; ^7^ Laboratory for Movement Biomechanics, Institute for Biomechanics, ETH Zurich, Zurich, Switzerland

**Keywords:** reactive balance training, falls, efficacy, dynamic stability, margin of stability, resilience

## Abstract

**Introduction:**

Single-session perturbation-based balance training (PBT) has demonstrated improvements in dynamic stability during the initial step following perturbation in older adults. However, its broader effects on comprehensive balance recovery remain inconclusive. This pilot laboratory-based randomized controlled study investigated the impact of personalized single-session PBT on reactive balance control during walking, employing advanced stability analysis techniques.

**Methods:**

Ten participants in the training group (67.1 ± 2.8 years; 5 males & 5 females) underwent a single session consisting of 32 unpredictable treadmill-induced slips and trips of progressively increasing intensity, while ten participants in the control group (72.8 ± 5.2 years; 5 males & 5 females) engaged in unperturbed treadmill walking. Key outcome measures included margin of stability (MoS) parameters: minimum MoS and the number of recovery steps, and resilience parameters: peak instability and recovery time, assessed at baseline, immediately post-intervention, and 3 months post-intervention following an unexpected treadmill slip.

**Results:**

In the training group, participants exhibited significant increases in minimum MoS values immediately post-intervention (−33 ± 84 mm; *p* < 0.001) and 3 months post-intervention (−71 ± 70 mm; *p* < 0.01) as compared to pre-intervention (−140 ± 87 mm); they also showed a significant reduction in peak instability immediately post-intervention (34 ± 14 mm; *p =* 0.019) as compared to pre-intervention (57 ± 25 mm). These changes were not observed in the control group. However, neither group demonstrated significant alterations in the number of recovery steps or recovery time across the different assessment periods.

**Discussion and conclusion:**

In conclusion, single-session PBT enhanced reactive balance control by improving the magnitude of post-perturbation responses, but it did not significantly influence the speed of recovery to baseline conditions.

## 1 Introduction

Falls and fall-related injuries are common health problems among older adults (Terroso et al., 2014; Mansfield et al., 2015; Salari et al., 2022; Niemann et al., 2023), and carry a significant societal cost (World Health Organization, 2021; Duc et al., 2022). Balance and functional exercises (e.g., sit-to-stand, stepping) can reduce fall incidence in community-dwelling older adults by around 24% (Sherrington et al., 2019), and are a key part of recommended guidelines for fall prevention (Montero-Odasso et al., 2022). However, these exercises primarily target older adults’ volitional balance control and have less focus on the reactive balance control (Wang et al., 2022). Volitional balance control involves intentional and feedforward movements to maintain stability. In contrast, reactive balance control involves automatic responses to unpredictable perturbations and relies on feedback mechanisms. This type of control is crucial for quickly recovering from the slips or trips, which have been the leading cause of falls during outdoor activities in community-dwelling older adults (Luukinen et al., 2000). Over the past two decades, perturbation-based balance training (PBT) has emerged as a promising fall-prevention method designed to enhance reactive balance control (McCrum et al., 2022). Through PBT, individuals learn to react and adapt to sudden losses of balance, gradually developing effective balance recovery strategies (McCrum et al., 2022). Importantly, PBT aligns with recent guidelines recommending balance-challenging exercises to prevent falls in community-dwelling older adults, emphasizing the need for more high-quality evidence (Montero-Odasso et al., 2022).

Traditionally, PBT is conducted over multiple sessions and has been shown to enhance reactive balance control and reducing fall incidence by 22%–44% (Rogers et al., 2021; Nørgaard et al., 2023). However, multi-session programs can be time-consuming and may suffer from low adherence rates (Pai et al., 2014a). Recent studies have explored whether delivering multiple perturbations within a single session could offer similar benefits. One study reported a 50% reduction in fall incidence following single-session PBT (Pai et al., 2014a), suggesting this approach may reduce the need for prolonged exercise and improve engagement. Research on single-session PBT has primarily focused on two key outcomes. In terms of real-life falls, a single session of 24 overground slips significantly reduced falls over 1 year (Pai et al., 2014a), whereas a single session of 40 treadmill slips did not lower the 6-month fall incidence (Wang et al., 2022). Regarding dynamic stability, studies often use the “margin of stability” (MoS) (Pai et al., 2014b; Lee et al., 2018; Wang et al., 2019a; Wang et al., 2019b; Wang et al., 2020; Bhatt et al., 2021; Liu et al., 2021; Song et al., 2021) to assess the effects of single-session PBT on reactive balance control, typically during the first recovery step after an unexpected perturbation (Pai et al., 2014b; Lee et al., 2018; Wang et al., 2019a; Wang et al., 2019b; Wang et al., 2020; Liu et al., 2021). A greater positive MoS reflects improved gait stability (Watson et al., 2021), with both immediate (Lee et al., 2018; Wang et al., 2019a; Wang et al., 2019b; Wang et al., 2020; Bhatt et al., 2021; Song et al., 2021) and long-term (Pai et al., 2014b; Liu et al., 2021) benefits observed in several studies. In summary, while single-session PBT improves dynamic stability, its impact on real-life fall reduction remains inconsistent. This may be attributed to small sample sizes in most previous studies, as fall incidence studies require large-scale trials for reliable results. Variations in training intensity and duration may also play a role (Pai et al., 2014a; Wang et al., 2022). Additionally, analyzing only the first recovery step might overlook some important aspects of fall prevention, suggesting the need for broader assessments in future research.

Exploration of the optimal training dose and personalized protocols for single-session PBT has been limited. Training doses play a critical role - doses that are too low may be ineffective in reducing fall risk, while doses that are too high could lower adherence and may not provide additional benefits in fall prevention beyond a certain threshold (Karamanidis et al., 2020). Most previous studies have implemented a fixed number of perturbations, constant perturbation intensity, and standardized scenarios for single-session PBT (Pai et al., 2014a; Pai et al., 2014b; Wang et al., 2019a; Wang et al., 2020; Bhatt et al., 2021; Liu et al., 2021; Song et al., 2021; Wang et al., 2022). Only two studies have investigated how the dose of single-session PBT influenced dynamic stability and reactive balance control (Lee et al., 2018; Wang et al., 2019b). Lee et al. found that 40 treadmill slips did not produce better immediate outcomes compared to 24 slips (Lee et al., 2018). In contrast, Wang et al. demonstrated that single-session PBT with progressively increasing intensities led to better immediate outcomes than a control group performing unperturbed treadmill walking (Wang et al., 2019b). Furthermore, few studies have incorporated enriched perturbation scenarios (such as a combination of slips and trips) within single-session PBT. Tailoring the training dose and incorporating diverse perturbation scenarios could provide a more comprehensive training experience, potentially enhancing fall prevention outcomes.

Another research gap is that analyzing the immediate response of dynamic stability after a perturbation may not fully capture the entire balance recovery and fall-avoidance mechanism. Measuring the minimum MoS following an unpredictable perturbation provides critical insight into the most unstable state during recovery (McCrum et al., 2018). Additionally, calculating the number of steps needed to return to baseline MoS helps assess recovery speed (McCrum et al., 2018; Debelle et al., 2020). Several advanced non-linear analysis techniques have also been proposed to evaluate balance control performance. Unlike traditional linear methods (e.g., standard deviation), which interpret variability around the mean as random noise, non-linear analysis suggests that fluctuations in normal human walking reflect patterns indicative of complex adaptive behavior (Stergiou, 2018). Ravi et al. introduced a method to assess steady-state behavior by considering the natural variability in walking patterns^28^. This approach also evaluates resilience - defined as the ability to resist perturbations or recover to steady state following a disturbance (Ravi et al., 2021a; Ravi et al., 2021b). Two key indicators of resilience can be derived: peak instability, defined as the maximum deviation from steady-state behavior following a perturbation, and recovery time, defined as the duration from peak instability to the return to steady state (Ravi et al., 2021a). By evaluating both the magnitude of the response (e.g., minimum MoS, minimum MoS relative to baseline, peak instability) and the speed of recovery (e.g., number of recovery steps, recovery time), we can gain deeper insights into the effects and mechanisms underlying single-session PBT. This approach can also help identify key factors that influence the success or failure of this type of training.

Given the identified research gaps, the primary objective of this study was to investigate the immediate and retained effects of single-session PBT on reactive balance control in older adults. The training incorporated enriched perturbation scenarios, featuring both treadmill slips and trips, with progressive intensities organized in blocks. Resilience and anteroposterior MoS parameters were evaluated following an unpredictable treadmill slip, comparing older adults who received the single-session PBT to those who did not, across different time points (i.e., pre-intervention, post-intervention, and 3 months post-intervention). It was hypothesized that: 1) Single-session PBT would result in greater reductions in peak instability, recovery time, and the number of recovery steps, along with greater increases in minimum MoS values and minimum MoS values relative to baseline from pre- to post-intervention, compared to the control group. 2) These improvements would persist at 3 months post-intervention compared to pre-intervention. The secondary objective was to determine whether single-session PBT could enhance balance performance, as measured by clinical tests, and reduce the incidence of prospective falls in older adults. It was hypothesized that the training group would show better clinical test results and experience fewer falls following the single-session PBT compared to the control group.

## 2 Methods

### 2.1 Study design and participants

This pilot laboratory-based assessor-blinded randomized controlled study was conducted at ETH Zurich, Switzerland, from June 2022 to June 2023. The study protocol has obtained ethical approval from the ETH ethics committee (Ethics Number: 2021-N-90). The flow of intervention and assessment procedures is displayed in Supplementary Figure S1. The study was performed in accordance with the Declaration of Helsinki.

Participants were recruited through convenience sampling using flyers and mail at local physiotherapy centers, the local ergotherapy association, the general practitioner association, and the ETH pensioner’s association. The inclusion criterion was community-dwelling people aged 65 years or older. The exclusion criteria were: a) diagnosed with acute or chronic musculoskeletal or neurological impairments, b) having undergone any lower limb surgery such as joint replacement, within the past 1.5 years, c) regular intake of antidepressants, and d) inability to walk on a treadmill for 30 min. All participants provided written informed consent. The participants were randomly allocated to the training or control group in a 1:1 ratio. Demographic data, including each participant’s age, sex, height, weight, leg dominance, and fall history within past 1 year were collected. Specifically, each participant was asked about the number of falls they had experienced in the past 12 months. A fall was defined as an unintentional event that led to the person coming to the ground or a lower level (World Health Organization, 2021). Fallers were defined as the people who experienced at least one fall in the past 12 months. Recurrent fallers were defined as the people who experienced two or more falls in the past 12 months. Each participant’s dominant leg was identified as their self-reported preferred leg used to kick a ball (Hoffman et al., 1998).

### 2.2 Equipment and intervention

A commercially available single-belt treadmill with an overhead safety harness (Quasar, H/P/Cosmos Sports & Medical GmbH, Nussdorf-Traunstein, Germany) was used for treadmill walking. A 12-camera motion capture system (Vicon T160, Vicon Motion Systems, Oxford, United Kingdom), sampling at 200 Hz, was used for kinematic data collection. A total of 62 reflective markers were affixed to the participant’s whole-body anatomical landmarks (see Supplementary Table S1). The treadmill was synchronized with the motion capture system to induce unpredictable perturbations. Specifically, a customized MATLAB script identified heel strikes (defined as the minimal vertical foot velocity per gait cycle) in real time (O’Connor et al., 2007). The heel strike data were then transmitted to Visual Studio, where a custom script sent a trigger to the treadmill, causing it to accelerate/decelerate the belt immediately following a detected heel strike. The perturbation occurred during the single-stance phase.

Prior to the intervention, the maximum treadmill belt accelerations, which challenged each participant’s forward and backward limits of standing stability without requiring a step, were determined by gradually increasing the treadmill belt acceleration in 0.25 m/s^2^ increments. The participant’s self-selected comfortable walking speed was determined for both the control and intervention groups, by gradually increasing the treadmill belt speed in 0.2 km/h increments. The final speed was calculated as the average of the speeds at which the participant reported feeling slightly too fast and slightly too slow. The details of participants’ comfortable walking speeds and limits of standing stability are presented in Supplementary Table S2.

#### 2.2.1 Personalized single-session PBT (training group)

Each participant completed a single session of PBT during treadmill walking at a comfortable speed. The session consisted of three eight-minute trials, with two-minute intervals for rest between trials. The perturbations included equal numbers of treadmill trips (belt acceleration) and slips (belt deceleration), randomly delivered to either foot. Each participant experienced 8 unpredictable perturbations in the first trial, 16 in the second trial, and another 8 in the third trial. The intensity of the perturbations progressively increased across the trials. In the first trial, the perturbation intensities of treadmill trips and slips were set at 1.25 times the previously determined forward and backward limits of standing stability, respectively. If the participant felt comfortable with the intensity and no fall occurred, the perturbation intensity was increased by 0.5 m/s^2^ for the next trial (Supplementary Table S2).

#### 2.2.2 Unperturbed walking (control group)

Each participant walked on the treadmill at a comfortable speed without any perturbations. This also comprised three eight-minute trials, with two-minute rest intervals between trials.

### 2.3 Primary outcome measures

Each participant’s reactive balance control during treadmill walking was assessed pre-intervention, immediately post-intervention, and 3 months post-intervention (Supplementary Figure S1). During each assessment, the participant only received one random treadmill slip (i.e., 2.25 m/s^2^ belt deceleration on the dominant leg) while walking. The participant was instructed not to talk, scratch, or adjust clothes during the treadmill walking, while the whole-body kinematics were collected. The primary outcome measures, namely, MoS and resilience, were processed and analyzed to quantify each participant’s reactive balance control performance during treadmill walking.

Before the calculation of MoS and resilience, the three-dimensional coordinates of all reflective markers were first low-pass filtered (Butterworth, zero-phase lag, fourth-order, 5Hz cut-off frequency). The sacrum marker was then used as an approximate representation of CoM (Yang and Pai, 2014).

#### 2.3.1 MoS

The MoS in the anteroposterior direction was calculated at each foot strike using the equations provided below (Hof et al., 2005; Curtze et al., 2024). The heel strikes identified in real time during treadmill walking were initially used as foot strikes (O’Connor et al., 2007). If the participant did not land on the heel following an unpredictable perturbation, motion capture data were checked in Vicon Nexus software to manually confirm the foot strike time points.

$$\text{MoS}={\text{BoS}}_{\;\text{anterior}}-\text{xCoM}$$ (1)

$$\text{xCoM}=x+\frac{v_{x}}{\omega_{0}}$$ (2)

$$v_{x}=v_{\text{CoM}}+\left|v_{T}\right|$$ (3)

$$\omega_{0}=\sqrt{\frac{g}{l}}$$ (4)

Briefly, the anteroposterior MoS was the difference between the anterior boundary of the base of support (or BoS_anterior_) and the extrapolated CoM position (or xCoM) (Equation 1, Supplementary Figure S2). The BoS_anterior_ was defined as the position of the toe marker of the leading leg in the anteroposterior direction (McCrum et al., 2018). The xCoM represented the anteroposterior extrapolated CoM position (Equation 2). Here, x was anteroposterior position of the CoM, and v_x_ was anteroposterior CoM velocity relative to the treadmill belt, which equaled the sum of the anteroposterior CoM velocity relative to ground and the absolute value of treadmill belt velocity (Equation 3). The ω_0_ was eigenfrequency of pendulum, the g was gravitational acceleration, and l was pendulum length which equaled the distance between CoM position and midpoint of the line connecting medial malleolus marker and lateral malleolus marker (McCrum et al., 2014) (Equation 4).

MoS values at foot strikes for the 10 consecutive steps before and 20 steps after the perturbed step were analyzed (Supplementary Figure S2). The baseline MoS was defined as the mean MoS values from the 10 steps prior to the perturbed step. The minimum MoS was obtained to represent the most unstable post-perturbation gait relative to zero (McCrum et al., 2018). Its positive sign indicated a stable gait, meaning the xCoM was within the BoS (Watson et al., 2021). The minimum MoS relative to baseline was further determined by taking the minimum MoS minus the baseline MoS, dividing by the baseline MoS, and multiplying by 100%. It represented the most unstable post-perturbation gait relative to pre-perturbation walking. The number of recovery steps was calculated as the number of steps from the one with the minimum MoS to the first step where the MoS returned to within one standard deviation (SD) below the baseline MoS (Debelle et al., 2020).

#### 2.3.2 Resilience

A participant’s resilience in reactive balance control was quantified by performing state space reconstruction of vertical CoM time series, to determine the pre-perturbation steady state boundaries and evaluate the post-perturbation CoM response (Ravi et al., 2021a). The processing procedures are detailed below.

The pre-perturbation and post-perturbation vertical CoM time series were reconstructed separately in state space using the time-delay embedding procedure (Wurdeman, 2018), which involved the determination of a time lag τ and an embedding dimension d. The resulting state space vectors [X(t), X(t+τ), …, X(t+(d-1)∗τ)] were further reduced to three dimensions [X(t), X(t+τ), X(t+2∗τ)]. The mean of all vectors on the reconstructed pre-perturbation trajectory was taken as the centroid, and a reference trajectory (M) was fitted to these vectors using an eight-term Fourier model. An ellipse was constructed around M at each integer angle ranging from 0° to 359°. The semi-major and semi-minor axes of the ellipse were set to twice the largest and second-largest SDs, respectively, as calculated from the 50 nearest vectors in three dimensions. All ellipses together formed a torus (T_2σ_), representing the boundaries of steady-state behavior (Supplementary Figure S3). Then the Euclidean distance, D(t), from each vector on the reconstructed post-perturbation trajectory to M was calculated (Supplementary Figure S3). The peak instability, defined as the maximum Euclidean distance, was analyzed to indicate the most unstable state following an unpredictable perturbation. Higher peak instability values reflect larger deviations from steady-state walking, indicating poorer balance control. The recovery time was defined as the time interval from the peak instability point to the recovery point, which was identified when the trajectory re-entered the torus (T_2σ_) and remained within it for three consecutive gait cycles, allowing for up to five outliers. Both this criterion and a more stringent criterion (allowing one outlier for five consecutive gait cycles) were tested. The current criterion was ultimately chosen, as it produced more reasonable recovery time values (see Supplementary Table S3).

### 2.4 Secondary outcome measures

Clinical tests were conducted to assess each participant’s postural balance performance (Supplementary Figure S1). The Timed Up and Go (TUG) test was performed pre-intervention and again 3 months post-intervention to evaluate volitional balance control. Participants were instructed to “stand up from the chair, walk a 3-m distance, walk back, and sit down,” with the completion time recorded (Podsiadlo and Richardson, 1991). An armless chair with back support was used in the TUG test. The Functional Reach Test (FRT) was conducted pre-intervention, immediately post-intervention, and 3 months post-intervention to evaluate volitional balance control. Participants were instructed to “raise the arm, make a fist, and reach forward as far as possible without making a step,” with the reached distance measured (Barker et al., 2014). The Push and Release (P&R) test was administered pre-intervention, immediately post-intervention, and 3 months post-intervention to evaluate reactive balance control. Participants were instructed to “lean backward against the examiner’s hands placed on their scapula”; the examiner then suddenly removed their hands to induce a stepping response, and the participant’s performance was rated using a specific scale (Valkovič et al., 2008). The longer completion time of TUG test, smaller reaching distance of FRT, and higher score of P&R test indicate poorer balance performance.

Monthly phone calls were conducted to follow up each participant’s number of prospective falls over the 6 months following the intervention (Supplementary Figure S1). Prospective fallers were defined as the people who had at least one fall during the tracked 6 months. Prospective multiple fallers were defined as the people who had two or more falls during the tracked 6 months.

### 2.5 Statistical analyses

Data of demographics and prospective falls were compared in training vs. control groups as below. For continuous variables (i.e., age, body mass index [BMI], number of previous falls, and number of prospective falls), independent sample t-tests were used when data were normally distributed, while Mann-Whitney U tests were used when data were not normally distributed. For discrete/dichotomous categorical variables (i.e., sex, dominant leg, fall history status, and prospective fall status), Fisher’s exact tests were used considering the small sample size.

Effects of “intervention” (between-subjects factor: training group vs. control group) and “time” (within-subjects factor: pre-, immediately post-, and 3 months post-intervention) on the outcomes of postural balance performance were examined as below. For each continuous outcome (i.e., the completion time of TUG test, the reached distance in FRT, the minimum MoS, the minimum MoS relative to baseline, the number of recovery steps, the peak stability, or the recovery time), a two-way mixed analysis of variance (ANOVA) followed by pairwise comparisons with Bonferroni corrections was conducted. The interaction effect of “intervention” and “time”, the main effect of each factor, and the simple effect of each factor were examined. Effect sizes (*f*) of interaction effects for primary outcome measures were calculated. The *f* values of 0.1, 0.25, and 0.4 represented small, medium, and large effect sizes, respectively (Cohen, 2013). For the ordinal outcome (i.e., the score of P&R test), a Mann-Whitney U test was used to compare group difference at each assessment, and a Friedman test followed by *post hoc* pairwise comparisons with Bonferroni corrections was conducted to examine the changes over time within each group. Extreme values were identified as the data points falling outside of 3 times the interquartile range from either the first or the third quartile (Seo, 2006). Statistical analyses were separately conducted to examine the data with and without the extreme values removed.

Post hoc correlation analyses were conducted between clinical test outcomes and primary outcomes for each time point of assessment. A Pearson correlation test was used if the two continuous variables followed normal distributions; otherwise, a Spearman correlation test was used. All statistical analyses were conducted in SPSS version 25.0 with the two-tailed significance level set at 0.05.

## 3 Results

Twenty-two eligible older adults were recruited for this study initially. One participant was excluded due to technical issues, and another one participant was excluded due to developing a secondary health problem during the follow-up. In the end, 20 participants were included in the data analysis, with 10 in the training group and 10 in the control group. As presented in Table 1, participants in the training group were significantly younger than those in the control group by 5 years (*p <* 0.01). There were no significant differences between groups in body mass index (BMI), sex, leg dominance, fall history status, or the number of previous falls (*p >* 0.05). Given the significant difference in age between two groups, two-way mixed analysis of covariance (ANCOVA, “intervention” × “time”) was also conducted for each primary outcome, with” “age” as the covariate, followed by pairwise comparisons with Bonferroni corrections.

**TABLE 1:** Results of demographics, prospective falls, and clinical tests.

Characteristics	Training group (n = 10)	Control group (n = 10)
Age (year)	67.1 ± 2.8*	72.8 ± 5.2*
BMI (kg/m^2^)	23.6 ± 3.0	22.8 ± 2.4
Female (number, percentage)	5, 50%	5, 50%
Right leg dominance (number, percentage)	8, 80%	10, 100%
Fall history status		
Fallers (number of participants, percentage)	2, 20%	5, 50%
Recurrent fallers (number of participants, percentage)	1, 10%	4, 40%
Frequency of previous falls	0 (0)	1 (2)
Prospective fall status		**n = 8**
Prospective fallers (number of participants, percentage)	3, 30%	3, 37.5%
Prospective multiple fallers (number of participants, percentage)	2, 20%	1, 12.5%
Frequency of prospective falls	0 (1)	0 (1)
TUG test (s)		
Pre-intervention	8.2 ± 1.4	8.6 ± 1.4
Three months post-intervention	8.2 ± 1.1	9.3 ± 2.1
FRT (cm)		
Pre-intervention	30.8 ± 3.6^#^	28.7 ± 6.9
Immediately post-intervention	29.5 ± 6.3^†^	26.1 ± 6.6
Three months post-intervention	38.7 ± 4.5^#†^*	31.5 ± 7.0*
P&R test (score)		
Pre-intervention	0 (1.0)	1.0 (1.0)
Immediately post-intervention	0.5 (1.0)	1.0 (2.0)
Three months post-intervention	0.5 (1.0)	1.0 (0)

Notes: Normally distributed continuous data were displayed as mean ± standard deviation. Non-normally distributed continuous data or ordinal data were displayed as median (interquartile range). BMI: body mass index. Fallers: people with ≥1 previous fall. Recurrent fallers: people with ≥2 previous falls. Prospective fallers: people with ≥1 prospective fall. Prospective multiple fallers: people with ≥2 prospective falls. TUG: timed up and go. FRT: functional reach test. P&R: push and release.

*Represents the significant difference between training group and control group (*p <* 0.05). # or † represents the significant change over time, and the same symbols represent the pairwise comparison with significant difference (*p <* 0.05).

### 3.1 MoS

**FIGURE 1:**
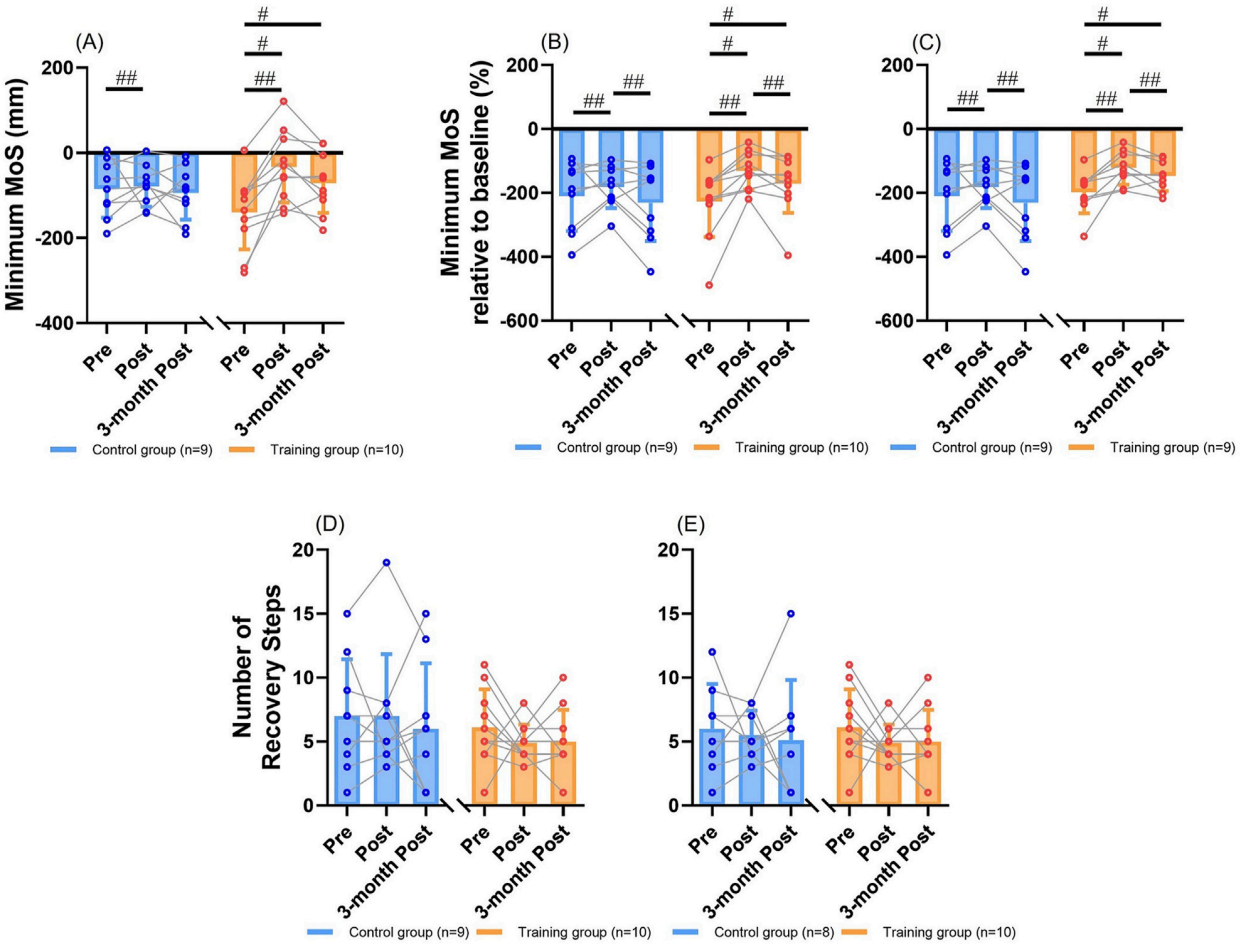
The MoS outcomes (mean ± SD) with individual values for comparisons between groups and among time points. **(A)** Results of the minimum MoS. **(B)** Results of the minimum MoS relative to baseline. **(C)** Results of the minimum MoS relative to baseline after excluding the extreme value. **(D)** Results of the number of recovery steps. **(E)** Results of the number of recovery steps after excluding the extreme value. # represents the significant change over time in a certain group (simple effect of “time”, *p <* 0.05). ## represents the significant change over time for both groups (main effect of “time”, *p <* 0.05). MoS: margin of stability. SD: standard deviation.

One participant in the control group held the handrails of treadmill for balance recovery following the unpredictable perturbations during assessment trials. This participant was excluded from the MoS analysis (Figure 1), as the calculation of MoS assumes the absence of external forces other than gravity and ground reaction forces (Curtze et al., 2024).

#### 3.1.1 Minimum MoS

Two-way mixed ANOVA revealed that there was a significant main effect of “time” (Figure 1A), showing that participants of two groups had generally increased minimum MoS values immediately post-intervention than pre-intervention (*p <* 0.01). There was no significant main effect of “intervention” (*p* > 0.05). There was a significant interaction effect of “intervention” and “time” (*p <* 0.01; *f* = 0.64). Significant simple effect of “time” existed within the training group (Figure 1A), where participants’ minimum MoS values significantly increased immediately post-intervention (−33 ± 84 mm [mean ± SD; same as below]; *p <* 0.001) and 3 months post-intervention (−71 ± 70 mm; *p <* 0.01) as compared to pre-intervention (−140 ± 87 mm). No significant simple effect of “time” was observed within the control group (pre-intervention: −85 ± 67 mm, immediately post-intervention: −78 ± 48 mm, three-month post-intervention: −94 ± 62 mm; *p >* 0.05). There was no significant simple effect of “intervention” at any time point (*p >* 0.05).

Two-way mixed ANCOVA identified the same significant results with those of ANOVA. There was a significant main effect of “time”, showing that participants of two groups had generally increased minimum MoS values immediately post-intervention than pre-intervention (*p* < 0.01). There was no significant main effect of “intervention” (*p* > 0.05). There was a significant interaction effect of “intervention” and “time” (*p* < 0.01; *f* = 0.59). Significant simple effect of “time” existed within the training group, where participants’ minimum MoS values significantly increased immediately post-intervention (*p <* 0.01) and 3 months post-intervention (*p <* 0.01) as compared to pre-intervention. No significant simple effect of “time” was observed within the control group (*p >* 0.05). There was no significant simple effect of “intervention” at any time point (*p >* 0.05).

#### 3.1.2 Minimum MoS relative to baseline

Two-way mixed ANOVA identified that there was a significant main effect of “time” (Figure 1B), showing that participants of two groups had generally larger values immediately post-intervention than pre-intervention (*p <* 0.01) or three-months post-intervention (*p =* 0.028). There was no significant main effect of “intervention” (*p >* 0.05). There was a significant interaction effect of “intervention” and “time” (*p* = 0.035; *f* = 0.47). Significant simple effect of “time” existed within the training group (Figure 1B), where participants’ minimum MoS values relative to baseline significantly increased immediately post-intervention (−131% ± 58%; *p <* 0.001) and 3 months post-intervention (−172% ± 90%; *p <* 0.01) as compared to pre-intervention (−227% ± 111%). No significant simple effect of “time” was observed within the control group (pre-intervention: −210% ± 109%, immediately post-intervention: −182% ± 65%, three-month post-intervention: −231% ± 119%; *p >* 0.05). There was no significant simple effect of “intervention” at any time point (*p >* 0.05). The identified significant results remained after excluding one participant in the training group who had extremely small minimum MoS relative to baseline (Figure 1C).

Two-way mixed ANCOVA revealed that there was a significant main effect of “time”, showing that participants of two groups had generally larger values immediately post-intervention than pre-intervention (*p =* 0.01) or three-months post-intervention (*p =* 0.016). There was no significant main effect of “intervention” (*p >* 0.05). There was a significant interaction effect of “intervention” and “time” (*p* = 0.035; *f* = 0.48). Significant simple effect of “time” existed within the training group, where participants’ minimum MoS values relative to baseline significantly increased immediately post-intervention (*p* = 0.017) and 3 months post-intervention (*p <* 0.01) as compared to pre-intervention. Significant simple effect of “time” was also observed within the control group, where participants’ minimum MoS values relative to baseline significantly decreased from immediately post-intervention to 3 months post-intervention (*p* = 0.026). There was no significant simple effect of “intervention” at any time point (*p >* 0.05). The identified significant results remained after excluding the extreme value in the training group.

#### 3.1.3 Number of recovery steps

Two-way mixed ANOVA showed that there were no significant main effects (*p >* 0.05), interaction effect (*p >* 0.05; *f* = 0.14), or simple effects of “intervention” and “time” (Figure 1D; *p >* 0.05). The training group had recovery steps of 6 ± 3, 5 ± 1, and 5 ± 2, while the control group had 7 ± 4, 7 ± 5, and 6 ± 5 at pre-intervention, immediately post-intervention, and 3 months post-intervention, respectively (Figure 1D). No significant results were found either, after excluding one participant in the control group who responded to the unpredictable perturbations by running and did not return to baseline BoS within 20 post-perturbation steps (Figure 1E; *p >* 0.05).

Similarly, two-way mixed ANCOVA did not identify any significant main effects (*p >* 0.05), interaction effect (*p >* 0.05; *f* = 0.14), or simple effects of “intervention” and “time” (*p >* 0.05) on the number of recovery steps. No significant results were found, either, after excluding the extreme value in the control group (*p >* 0.05).

### 3.2 Resilience

#### 3.2.1 Peak instability

**FIGURE 2:**
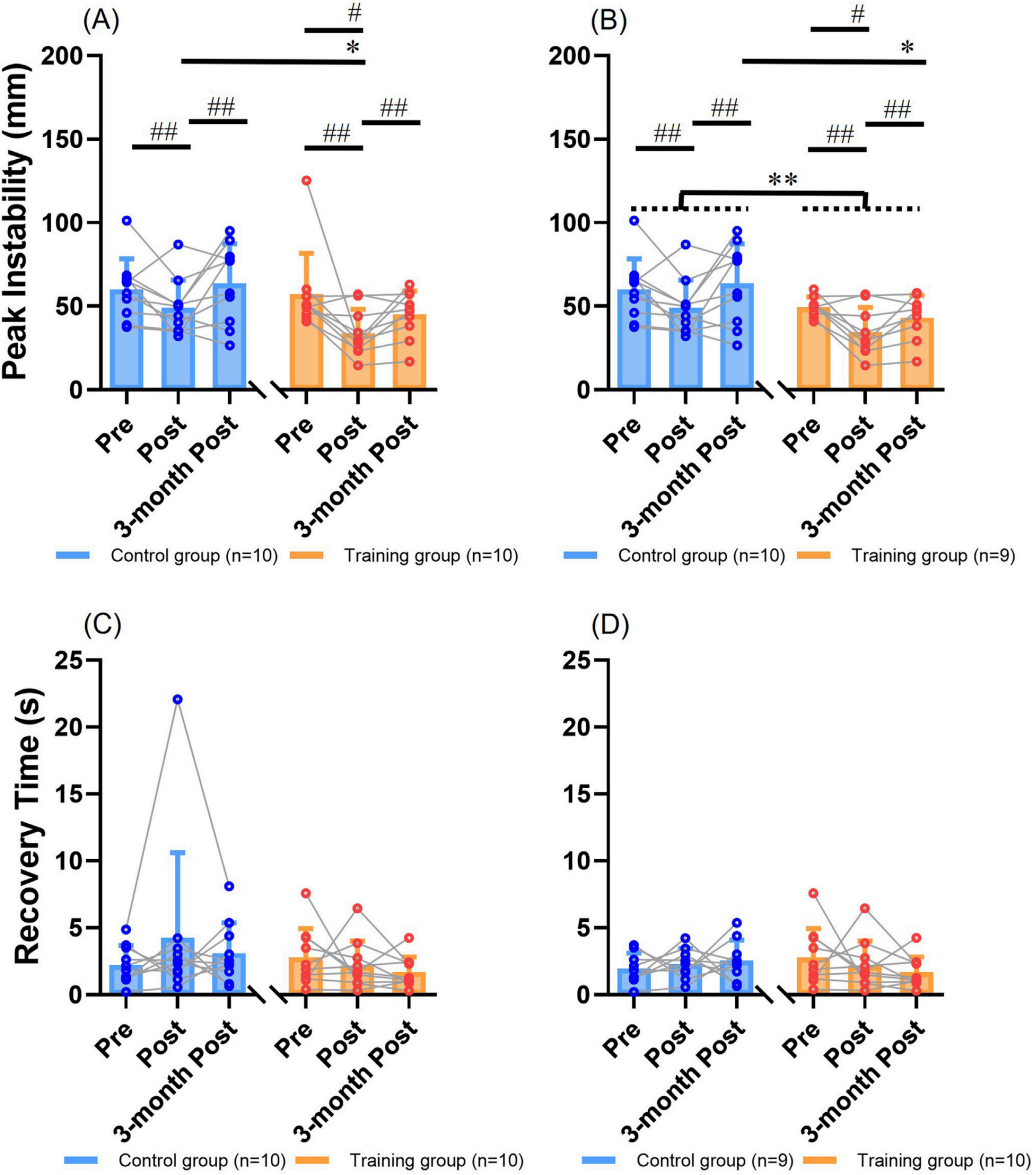
The resilience outcomes (mean ± SD) with individual values for comparisons between groups and among time points. **(A)** Results of the peak instability. **(B)** Results of the peak instability after excluding the extreme value. **(C)** Results of the recovery time. **(D)** Results of the recovery time after excluding the extreme value. # represents the significant change over time in a certain group (simple effect of “time”, *p <* 0.05). ## represents the significant change over time for both groups (main effect of “time”, *p <* 0.05). * represents the significant difference between training group and control group at a certain time point (simple effect of “intervention”, *p <* 0.05). ** represents the significant difference between training group and control group for all time points (main effect of “intervention”, *p <* 0.05). SD: standard deviation.

Two-way mixed ANOVA revealed that there was a significant main effect of “time” (Figure 2A), showing that participants of two groups had generally lower peak instability values immediately post-intervention than pre-intervention (*p =* 0.014) or three-months post-intervention (*p =* 0.017). There was no significant main effect of “intervention” (*p =* 0.086). No significant interaction effect of “intervention” and “time” was observed (*p >* 0.05; *f* = 0.29). The training group had peak instability values of 57 ± 25, 34 ± 14, and 45 ± 14 mm, while the control group had 60 ± 18, 49 ± 17, and 64 ± 24 mm at pre-intervention, immediately post-intervention, and 3 months post-intervention, respectively. Significant simple effect of “time” existed within the training group, where the peak instability values significantly decreased from pre-intervention to immediately post-intervention (*p =* 0.019, Figure 2A). No significant simple effect of “time” was observed within the control group (*p =* 0.078). Significant simple effect of “intervention” existed for immediate post-intervention, where participants in the training group showed significantly lower peak instability values than the control group (*p =* 0.044, Figure 2A). These significant results were still observed after excluding one participant in the training group who showed extremely high peak instability, except for the changes below (Figure 2B). Significant main effect of “intervention” was further observed, showing that the training group had generally lower peak instability values than the control group for all time points (*p =* 0.027, Figure 2B). Significant simple effect of “intervention” existed at 3 months post-intervention, where participants in the training group showed significantly lower peak instability values than the control group (Figure 2B, *p =* 0.038).

Two-way mixed ANCOVA revealed that there was a significant main effect of “time”, showing that participants of two groups had generally lower peak instability values immediately post-intervention than pre-intervention (*p =* 0.014) or three-months post-intervention (*p <* 0.01). There was no significant main effect of “intervention” (*p >* 0.05). No significant interaction effect of “intervention” and “time” was observed (*p >* 0.05; *f* = 0.33). Significant simple effect of “time” existed within the control group, where the peak instability value significantly increased from immediately post-intervention to 3 months post-intervention (*p <* 0.01). No significant simple effect of “time” was observed within the training group (*p >* 0.05). Significant simple effect of “intervention” existed at 3 months post-intervention, where participants in the training group showed significantly lower peak instability values than the control group (*p =* 0.037). These identified significant results remained after excluding the extreme value in the training group.

#### 3.2.2 Recovery time

Two-way mixed ANOVA revealed that there were no significant main effects (*p >* 0.05), interaction effect (*p >* 0.05; *f* = 0.28), or simple effects of “intervention” or “time” (Figure 2C; *p >* 0.05). The training group had recovery time of 2.8 ± 2.2, 2.2 ± 1.8, and 1.7 ± 1.2 s, while the control group had 2.2 ± 1.4, 4.3 ± 6.3, and 3.1 ± 2.3 s at pre-intervention, immediately post-intervention, and 3 months post-intervention, respectively (Figure 2C). No significant results were found either, after excluding the participant in the control group who responded by running following unpredictable perturbations and showed an extremely long time to return to steady state (Figure 2D; *p >* 0.05).

Similarly, two-way mixed ANCOVA did not identify any significant main effects (*p >* 0.05), interaction effect (*p >* 0.05; *f* = 0.24), or simple effects of “intervention” or “time” (*p >* 0.05) on the recovery time. No significant results were found either, after excluding the extreme value in the control group (*p >* 0.05).

### 3.3 Clinical test results and prospective falls

Clinical test results are summarized in Table 1. For the FRT, participants in the training group showed a significantly larger reaching distance 3 months post-intervention than pre-intervention or immediately post-intervention (*p <* 0.01), which was not observed in the control group. Additionally, participants in the training group showed a significantly larger reaching distance than those in the control group at three-month post intervention (*p =* 0.014). For the completion time of TUG test and the P&R score, no significant differences were observed between two groups or across three time points (*p >* 0.05).

The correlations between clinical test outcomes and primary outcomes were mostly non-significant (*p >* 0.05, Table 2). At pre-intervention, the longer completion time of TUG test was significantly related to higher minimum MoS (*r* = 0.596, *p* < 0.01) and higher minimum MoS relative to baseline following an unpredictable perturbation (*ρ* = 0.574, *p* = 0.01), and the smaller reaching distance of FRT was significantly related to fewer recovery steps following an unpredictable perturbation (*r* = 0.574, *p* = 0.01). At 3 months post-intervention, the larger score of P&R test was significantly related to the lower minimum MoS relative to baseline (*ρ* = −0.490, *p* = 0.033) and longer recovery time (*ρ* = 0.491, *p* = 0.028) following an unpredictable perturbation.

**TABLE 2:** Correlation test results between primary outcomes and clinical test outcomes.

Clinical tests	MoS parameters (n = 19)			Resilience parameters (n = 20)	
	Minimum MoS (mm)	Minimum MoS relative to baseline (cm)	Numbe*r* of recovery steps	Peak instability (mm)	Recovery time (s)
TUG test (s)					
Pre-intervention	*r* = 0.596**	ρ = 0.574*	*r* = −0.063	ρ = −0.083	ρ = 0.005
Three months post-intervention	ρ = 0.018	ρ = −0.030	ρ = −0.210	ρ = 0.059	ρ = 0.005
FRT (cm)					
Pre-intervention	*r* = −0.119	ρ = −0.089	*r* = 0.574*	ρ = −0.137	ρ = 0.120
Immediately post-intervention	*r* = 0.283	*r* = 0.218	ρ = −0.123	*r* = −0.377	ρ = −0.298
Three months post-intervention	*r* = 0.037	ρ = 0.135	ρ = 0.036	*r* = −0.034	ρ = −0.361
P&R test (score)					
Pre-intervention	ρ = 0.235	ρ = 0.115	ρ = 0.025	ρ = 0	ρ = −0.108
Immediately post-intervention	ρ = −0.267	ρ = −0.241	ρ = 0.155	ρ = 0.295	ρ = 0.118
Three months post-intervention	ρ = −0.453	ρ = -0.490*	ρ = 0.348	ρ = 0.25	ρ = 0.491*

Notes: The *r* is the Pearson correlation coefficient. The ρ is the Spearman correlation coefficient. The bold text and * represent *p* < 0.05. ** represents *p* < 0.01. TUG: timed up and go. FRT: functional reach test. P&R: push and release.

During the tracking of prospective falls, two participants in the control group were lost to follow-up. No significant differences were found in prospective fall status or the number of prospective falls between the two groups (Table 1, *p >* 0.05).

## 4 Discussion

The primary objective of this pilot randomized controlled study was to quantify how a single-session PBT, involving mixed slips and trips with progressive intensities, affected older adults’ reactive balance performance during walking immediately and in long term. Contrary to our hypotheses, not all of the examined outcomes exhibited improvements following the personalized single-session PBT. It enhanced older adults’ reaction magnitudes (i.e., minimum MoS, minimum MoS relative to baseline, and peak instability) in response to unpredictable perturbations. However, it did not significantly improve their recovery speed to pre-perturbation levels (i.e., number of recovery steps and recovery time).

Large effect sizes (f > 0.4) suggest that the single-session PBT had both immediate and lasting effects in improving minimum MoS values and their baseline-relative measures, as well as an immediate effect in reducing peak instability following unpredictable treadmill slips in older adults. The initial slip led to a reduction in step length, contributing to a lower MoS during the first few steps after the perturbation. This trend is consistent with previously reported stride-to-stride MoS responses following a perturbation (McCrum et al., 2018; Gerards et al., 2021). The improved minimum MoS parameters indicate that the single-session PBT enhanced dynamic stability during the most unstable phase of reactive balance control. This finding aligns with previous studies showing that single-session PBT increased dynamic stability during the first post-perturbation step (Wang et al., 2019b; Bhatt et al., 2021; Liu et al., 2021), although few studies have examined minimum MoS in stride-to-stride responses. Additionally, this study found that the single-session PBT reduced the peak deviation of the CoM from the steady state immediately after the intervention. Collectively, these findings on reaction magnitudes suggest that a single-session PBT can enhance older adults’ stability during the most unstable moments of gait or CoM displacement in reactive balance control.

The single-session PBT did not significantly reduce the number of recovery steps or the recovery time following unpredictable treadmill slips. Notably, previous studies have not directly shown that PBT accelerates balance recovery to pre-perturbation levels. Rather, they have highlighted participants’ adaptations in balance recovery speed (McCrum et al., 2018; Gerards et al., 2021) and muscle activation speed (Zhu et al., 2022; Tong et al., 2023; Zhu et al., 2025). As previously reported, young adults (McCrum et al., 2018) and older adults without a fall history (Gerards et al., 2021) were faster (i.e., taking fewer recovery steps to attain baseline MoS) in response to later unpredictable trips than to earlier ones. This discrepancy may be partly due to the greater number of experienced perturbations in this study (32 versus 8 perturbations) (McCrum et al., 2018; Gerards et al., 2021), which may have led to increased fatigue among participants, hindering their adaptation in recovery steps to baseline MoS and recovery time to return to CoM steady state. Future study may consider prescribing the individualized intensities of PBT based on a scale that quantifies the participant’s degree of fatigue, such as the rating of perceived exertion. Moreover, further investigation into how participants adapted to perturbations during the single-session training is warranted to optimize the appropriate dose of PBT. For example, if the number of recovery steps or recovery time plateaus as perturbation number or intensity increases, this may indicate that the dose is sufficient for the participant.

The effects of single-session PBT on resilience and dynamic stability remained, after adjusting for the confounding effects of age. Older adults were previously reported to have decreased margin of stability in response to a sudden backward perturbation (Martelli et al., 2017) or during obstacle crossing (Hak et al., 2019), as compared to young adults. In this pilot study, it is inferred that the improvements in balance control (i.e., increase of minimum MoS and decrease of peak instability) could be attributed to the significantly younger age of training group as compared to the control group. The ANCOVA was therefore conducted to account for the group age difference. Generally, the significant results identified by ANCOVA were similar to those of ANOVA. The major difference was that ANCOVA further identified the control group’s deterioration in the reaction magnitudes from immediately post-intervention to 3 months post-intervention (i.e., significant decrease of minimum MoS relative to baseline & significant increase of peak instability), which was not observed for ANOVA. With the age difference controlled, the ANCOVA findings confirmed that the personalized single-session PBT was primarily responsible for the participants’ improvements in balance control magnitude.

Although the single-session PBT produced similar responses in MoS and resilience parameters, it is important to note that these measures reflect different aspects of balance control. Both MoS and resilience parameters captured improvements in reaction magnitudes but did not show changes in recovery speed following unpredictable perturbations. However, there are key differences between the two measures that warrant further consideration. First, MoS represents how far the extrapolated horizontal displacement of the CoM is from the boundary of the BoS (Hof et al., 2005), whereas resilience is derived from the reconstruction of vertical CoM displacements and assesses how far the post-perturbation state deviates from the steady state (Ravi et al., 2021a). Second, MoS is typically calculated at specific time events (e.g., foot strike), representing instantaneous dynamic stability (Pai et al., 2014b; Lee et al., 2018; Wang et al., 2019a; Wang et al., 2019b; Wang et al., 2020; Bhatt et al., 2021; Liu et al., 2021; Song et al., 2021). In contrast, resilience is derived from state space reconstruction of adjacent vertical CoM displacement time series, capturing the variability of vertical CoM displacements over time (Ravi et al., 2021a). These differences may explain the varying effect sizes observed between MoS parameters (minimum MoS and baseline-relative MoS: large; number of recovery steps: small) and resilience parameters (peak instability: medium; recovery time: small) in quantifying the effects of the single-session PBT. The suspensory strategy, which involves lowering the CoM height, has been recognized as a key balance control mechanism (Kasahara and Saito, 2021; Tong et al., 2023; Zhu et al., 2025). Resilience parameters offer an additional nonlinear assessment of vertical CoM displacement, complementing prior linear analyses. When combined with MoS parameters, resilience measures may provide a more comprehensive evaluation of balance performance and the effects of training.

Contrary to our hypotheses for the secondary objective, not all clinical test results exhibited improvements, and there was no reduction in prospective falls following the personalized single-session PBT. The clinical test results suggest that the single-session PBT primarily enhanced the limits of volitional standing stability (as measured by the FRT), rather than influencing the number of reactive steps (P&R test) or volitional gait speed (TUG test). These results align with previous research demonstrating the effects of PBT on TUG test performance (Mansfield et al., 2010; Aviles et al., 2019; Allin et al., 2020; Lurie et al., 2020; Brüll et al., 2023). Although the impact of PBT on FRT performance has been reported in post-stroke individuals (Niewolak et al., 2022) and those with knee osteoarthritis (Bhaskar et al., 2019), evidence for its effectiveness in community-dwelling older adults remains limited. In this study, clinical test results have echoed the findings related to reaction magnitudes and recovery speed following unpredictable perturbations during walking, reinforcing the improvements of single-session PBT on reaction magnitudes. However, the single-session PBT did not lead to a reduction in prospective falls among older adults over the 6-month follow-up period. A previous study similarly found that a single session of treadmill slips in 70 older adults did not reduce prospective falls over a 6-month period (Wang et al., 2022), while another study reported that a single session of overground slips in 67 older adults led to a reduction in falls over 12 months (Pai et al., 2014a). The primary reason for these discrepancies may be the duration of fall tracking, as prospective falls tend to increase exponentially with aging and the accumulation of risk factors (Todd and Skelton, 2004). Additionally, factors such as training dose and sample size may have influenced the effectiveness of single-session treadmill-based PBT in preventing falls.

The MoS and resilience parameters may serve as valuable complements to existing tests for assessing balance control and fall risk in older adults. While prospective fall incidence remains the most direct measure of PBT effectiveness in fall prevention, tracking falls over time can be labour-intensive and slow. Clinical tests, such as the TUG test (where a completion time exceeding 15 s indicates fall risk) (Montero-Odasso et al., 2022), offer a quick way to identify at-risk individuals. However, these tests may lack the sensitivity needed to detect subtle changes in balance control, particularly in well-functioning community-dwelling older adults, like those in the current study. This study has identified some significant correlations between MoS/resilience parameters and the clinical test measures. A greater number of reactive steps (higher score of P&R test) was associated with the poorer dynamic stability (lower value of minimum MoS) and longer recovery time to steady-state walking. The completion time for TUG test was positively related to the minimum MoS value, possibly indicating that individuals with a slower walking speed might have better dynamic stability. The observed positive correlation between the reaching distance of FRT and the number of recovery steps might indicate that individuals with a larger forward limit of standing stability could withstand more steps where the xCoM exceeded beyond the anterior boundary BoS following an unexpected perturbation (i.e., more recovery steps to return to baseline MoS). However, it is worth noting that these significant relationships were not consistently observed across all three assessments, i.e., pre-, immediately post-, and 3 months post-intervention. Moreover, clinical test outcomes showed mostly non-significant correlations with MoS and resilience parameters, potentially suggesting that these measures assess different aspects of balance control. By supplementing clinical tests, MoS and resilience parameters may provide a more comprehensive evaluation of balance performance and the effects of single-session PBT.

The current study has several limitations. Firstly, we did not prospectively register this pilot study in a public clinical trial platform and the small sample size may have limited the ability to detect significant training effects from the single-session PBT. For example, although changes in recovery time after the intervention were not statistically significant, they showed a medium effect size (f = 0.28). Based on this effect size, a future study would require an estimated total of 36 older participants to achieve sufficient power (0.95) for detecting significant changes in recovery time, using a two-way mixed ANOVA design (two groups × three time points) at an α value of 0.05. Secondly, given the unmatched ages of training and control groups, this pilot study used ANCOVA to adjust for the age difference. Future RCTs may benefit from stratified randomization, categorizing participants into different age groups before randomly assigning them to the training or control groups within each stratum. Thirdly, discrepancies exist between the calculation of MoS in this study and the recommended methods (Curtze et al., 2024). Specifically, toe markers from both feet were used to define the anteroposterior boundaries of the BoS, as the treadmill used in this study could not record center-of-pressure (CoP) boundaries, as recommended (Curtze et al., 2024). Additionally, the sacrum marker was used as a proxy for the CoM to ensure comparability with resilience calculations (Ravi et al., 2021a; Ravi et al., 2021b), although ideally, the CoM shall be estimated using a whole-body kinematic model (Curtze et al., 2024). These limitations should be carefully considered when interpreting the findings of this pilot study. Fourthly, although this study has tried to induce the participants’ reactive balance control and minimize their anticipation by randomly inducing only one perturbation, future research might consider using electromyography to confirm if the potential anticipatory postural control exists (Bouisset and Do, 2008). Lastly, this study focused on the effects of PBT on physical abilities, without investigating the psychological effects. Fear of falling, low balance confidence, and fear of falling avoidance behaviors have been identified as the risk factors for future falls (Landers et al., 2016; Asai et al., 2022). Several prior studies have also investigated the effects of PBT on fear of falling (Kurz et al., 2016; Gerards et al., 2023) and balance confidence (Lurie et al., 2020); however, PBT showed limited effectiveness on them. On the one hand, future investigation is merited to examine whether the personalized single-session PBT in this study could increase fall efficacy or balance confidence, as the improvements in these psychological measures may further reduce the older people’s fear of falling avoidance behaviors and enhance the fall-prevention effects. On the other hand, these psychological measures shall be considered to inform the design or optimization of PBT (Soh, 2022).

In conclusion, after a single session of treadmill slip and trip training with progressively increasing intensities, older participants could exhibit improved dynamic stability and have reduced deviation from steady-state walking in response to the sudden slip. However, this training did not significantly improve the speed of recovery to unperturbed walking. Future studies with larger samples are necessary to confirm the findings of this pilot study.

## Data Availability

The original contributions presented in the study are included in the article/Supplementary Material, further inquiries can be directed to the corresponding author.
