# Supplementary material for: Effects of single-session perturbation-based balance training with progressive intensities on resilience and dynamic gait stability in healthy older adults
Source: Front Bioeng Biotechnol. 2025 Aug 29;13:1642158. doi: 10.3389/fbioe.2025.1642158 (PMC12426064; doi:10.3389/fbioe.2025.1642158)
Supplement: Supplementary file 1 [file DataSheet1.docx]

**Supplementary Table 1. Locations of reflective markers.**

| **Name** | **Segment** | **Location** |
| --- | --- | --- |
| RTO1 | Foot - right | 1st metatarsal head |
| RTO3 | Foot - right | 3rd metatarsal head |
| RTO5 | Foot - right | 5th metatarsal head |
| RHEE | Foot - right | Heel/Calcaneus |
| RMMA | Shank - right | Medial malleolus |
| RLMA | Shank - right | Lateral malleolus |
| RTMT | Shank - right | Tibia shaft, ventral |
| RTLF | Shank - right | Fibula shaft, lateral |
| RTTT | Shank - right | Tibial tuberosity |
| RTIB | Shank - right | Fibula head |
| RMCO | Thigh - right | Medial epicondyle of femur |
| RLCO | Thigh - right | Lateral epicondyle of femur |
| RTFR | Thigh - right | Femur, front distal |
| RTLL | Thigh - right | Femur, lateral distal |
| RTLH | Thigh - right | Femur, lateral proximal |
| RASI | Pelvis | Right ASIS (Anterior Superior Iliac Spine) |
| RTMS | Pelvis | Right iliac |
| RPSI | Pelvis | Right PSIS (Posterior Superior Iliac Spine) |
| SACR | Pelvis | Sacrum |
| LPSI | Pelvis | Left PSIS (Posterior Superior Iliac Spine) |
| LTMS | Pelvis | Left iliac |
| LASI | Pelvis | Left ASIS (Anterior Superior Iliac Spine) |
| LTO1 | Foot - left | 1st metatarsal head |
| LTO3 | Foot - left t | 3rd metatarsal head |
| LTO5 | Foot - left | 5th metatarsal head |
| LHEE | Foot - left | Heel/Calcaneus |
| LMMA | Shank - left | Medial malleolus |
| LLMA | Shank - left | Lateral malleolus |
| LTMT | Shank - left | Tibia shaft, ventral |
| LTLF | Shank - left | Fibula shaft, lateral |
| LTTT | Shank - left | Tibial tuberosity |
| LTIB | Shank - left | Fibula head |
| LMCO | Thigh - left | Medial epicondyle of femur |
| LLCO | Thigh - left | Lateral epicondyle of femur |
| LTFR | Thigh - left | Femur, front distal |
| LTLL | Thigh - left | Femur, lateral distal |
| LTLH | Thigh - right | Femur, lateral proximal |
| RWRA | Forearm - right | Styloid process of radius |
| RWUL | Forearm - right | Styloid process of ulna |
| RFRA | Forearm - right | Forearm, lateral/radial side |
| RFUL | Forearm - right | Forearm, medial/ulnar side |
| RMEC | Humerus - right | Medial epicondyle of humerus |
| RLEC | Humerus - right | Lateral epicondyle of humerus |
| RHLT | Humerus - right | Upper arm, lateral |
| RHVT | Humerus - right | Upper arm, ventral |
| RSHO | Shoulder | Right acromion |
| MSTC | Shoulder | Sternal notch |
| CVC7 | Shoulder | C7 |
| LSHO | Shoulder | Left acromion |
| LWRA | Forearm - left | Styloid process of radius |
| LWUL | Forearm - left | Styloid process of ulna |
| LFRA | Forearm - left | Forearm, lateral/radial side |
| LFUL | Forearm - left | Forearm, medial/ulnar side |
| LMEC | Humerus - left | Medial epicondyle of humerus |
| LLEC | Humerus - left | Lateral epicondyle of humerus |
| LHLT | Humerus - left | Upper arm, lateral |
| LHVT | Humerus – left | Upper arm, ventral |
| RFHD | Head | Right front of head |
| RBHD | Head | Right back of head |
| LFHD | Head | Left front of head |
| LBHD | Head | Left back of head |

**Supplementary Table 2. Participants’ comfortable walking speeds, limits of standing stability, and perturbation intensities for training.**

| **Group** | **Participant** | **Comfortable Walking Speed (m/s)** | **Limit of Standing Stability (m/s^2^)** | | **Perturbation Intensities for Training (m/s^2^)** | | |
| --- | --- | --- | --- | --- | --- | --- | --- |
|  |  |  | **Forward** | **Backward** | **Trial 1 (acc. & dec.)** | **Trial 2 (acc. & dec.)** | **Trial 3 (acc. & dec.)** |
| Training | #2 | 1.25 | 0.75 | 0.50 | 1.125 &  0.750 | 1.625 &  1.250 | 2.125 &  1.750 |
|  | #3 | 1.10 | 1.00 | 1.00 | 1.500 &  1.500 | 2.000 &  2.000 | 2.500 &  2.500 |
|  | #5 | 1.00 | 0.75 | 0.75 | 1.125 &  1.125 | 1.625 &  1.625 | 2.125 &  2.125 |
|  | #11 | 1.00 | 0.75 | 0.50 | 1.125 &  0.750 | 1.625 &  1.250 | 2.125 &  1.750 |
|  | #14 | 1.10 | 1.00 | 0.50 | 1.500 &  0.750 | 2.000 &  1.250 | 2.500 &  1.750 |
|  | #15 | 1.25 | 0.50 | 0.50 | 0.750 &  0.750 | 1.250 &  1.250 | 1.750 &  1.750 |
|  | #16 | 1.00 | 1.25 | 0.75 | 2.000 &  1.250 | 2.000 &  1.750 | 2.250 &  2.250 |
|  | #18 | 1.40 | 0.50 | 0.75 | 0.750 &  1.125 | 1.250 &  1.625 | 1.750 &  2.125 |
|  | #19 | 1.10 | 0.75 | 0.75 | 1.125 &  1.125 | 1.625 &  1.625 | 2.125 &  2.125 |
|  | #21 | 1.25 | 1.00 | 1.00 | 1.500 &  1.500 | 2.000 &  2.000 | 2.500 &  2.500 |
| Control | #1 | 1.35 | 0.75 | 0.50 | - | - | - |
|  | #4 | 1.20 | 0.50 | 0.75 | - | - | - |
|  | #6 | 1.25 | 1.00 | 0.50 | - | - | - |
|  | #7 | 1.25 | 1.25 | 0.75 | - | - | - |
|  | #8 | 1.10 | 0.75 | 0.75 | - | - | - |
|  | #9 | 1.45 | 0.75 | 0.75 | - | - | - |
|  | #10 | 1.40 | 1.00 | 0.75 | - | - | - |
|  | #12 | 0.90 | 0.75 | 0.50 | - | - | - |
|  | #17 | 1.50 | 0.75 | 0.50 | - | - | - |
|  | #20 | 1.30 | 1.00 | 1.00 | - | - | - |

Note: **acc.**: acceleration. **dec.**: deceleration.

**Supplementary Table 3. The recovery time values (s) based on two different criteria of identifying recovery points.**

| **Group** | **Participant No.** | **Pre-intervention** | | **Post-intervention** | | **Three months post-intervention** | |
| --- | --- | --- | --- | --- | --- | --- | --- |
|  |  | Loose | Stringent | Loose | Stringent | Loose | Stringent |
| Training | #2 | 2.245 | 2.265 | 3.845 | 4.110 | 2.335 | 2.390 |
|  | #3 | 3.485 | 10.390 | 2.740 | 2.765 | 0.870 | 1.345 |
|  | #5 | 1.185 | 1.185 | 0.905 | 0.905 | 0.965 | 0.965 |
|  | #11 | 1.065 | 6.760 | 6.455 | 6.515 | 2.295 | 2.335 |
|  | #14 | 1.890 | 1.910 | 2.145 | 2.145 | 2.425 | 2.440 |
|  | #15 | 7.565 | 31.450 | 0.850 | 0.850 | 0.280 | 11.885 |
|  | #16 | 0.390 | 0.425 | 0.295 | 0.345 | 1.020 | 1.020 |
|  | #18 | 4.320 | 4.360 | 1.475 | 1.490 | 1.100 | 1.120 |
|  | #19 | 4.260 | 4.285 | 1.690 | 11.475 | 4.235 | 4.310 |
|  | #21 | 1.470 | 6.025 | 1.660 | 1.660 | 1.265 | 1.265 |
| Control | #1 | 3.690 | 3.705 | 1.140 | 5.930 | 2.205 | 2.205 |
|  | #4 | 2.135 | 2.150 | 3.445 | 3.465 | 2.530 | 2.550 |
|  | #6 | 1.825 | 1.945 | 1.790 | 1.850 | 4.375 | 5.230 |
|  | #7 | 1.200 | 1.470 | 2.980 | 3.000 | 0.810 | 0.870 |
|  | #8 | 3.510 | 7.870 | 1.660 | 5.205 | 5.360 | 5.360 |
|  | #9 | 2.610 | 2.640 | 2.550 | 5.325 | 2.300 | 2.655 |
|  | #10 | 4.860 | 13.465 | 22.075 | 22.200 | 8.095 | 8.095 |
|  | #12 | 0.200 | 3.945 | 0.550 | 0.560 | 3.015 | 3.015 |
|  | #17 | 1.330 | 1.455 | 2.315 | 7.885 | 1.725 | 1.725 |
|  | #20 | 1.110 | 1.145 | 4.210 | 12.000 | 0.645 | 3.070 |

Note: The currently used loose criterion means that the recovery point remained within the torus (T_2σ_) for 3 consecutive gait cycles, allowing for up to 5 outliers. The stringent criterion means that the recovery point remained within the torus (T_2σ_) for 5 consecutive gait cycles, allowing for up to 1 outlier.


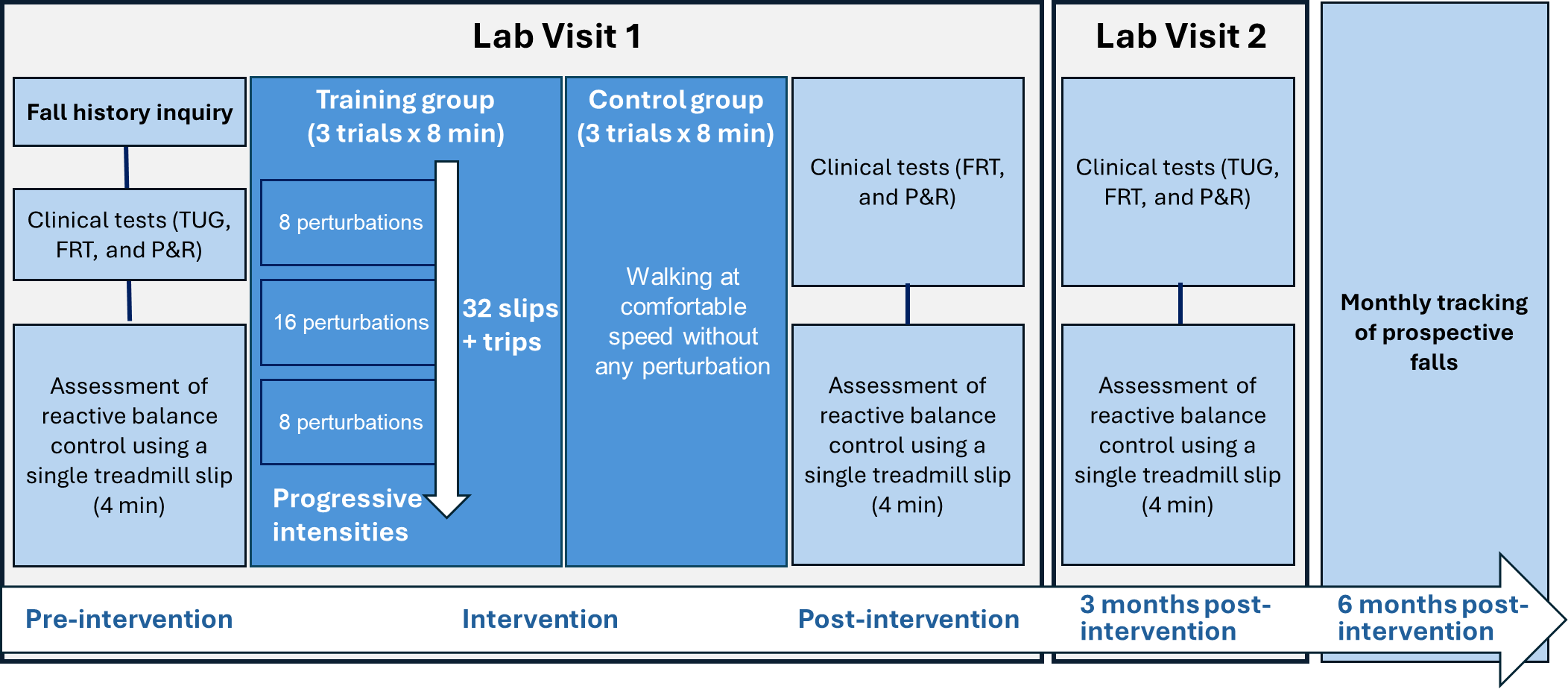


Supplementary Figure 1. Flowchart of intervention and assessment procedures. TUG: timed up and go test. FRT: functional reach test. P&R: push and release test.


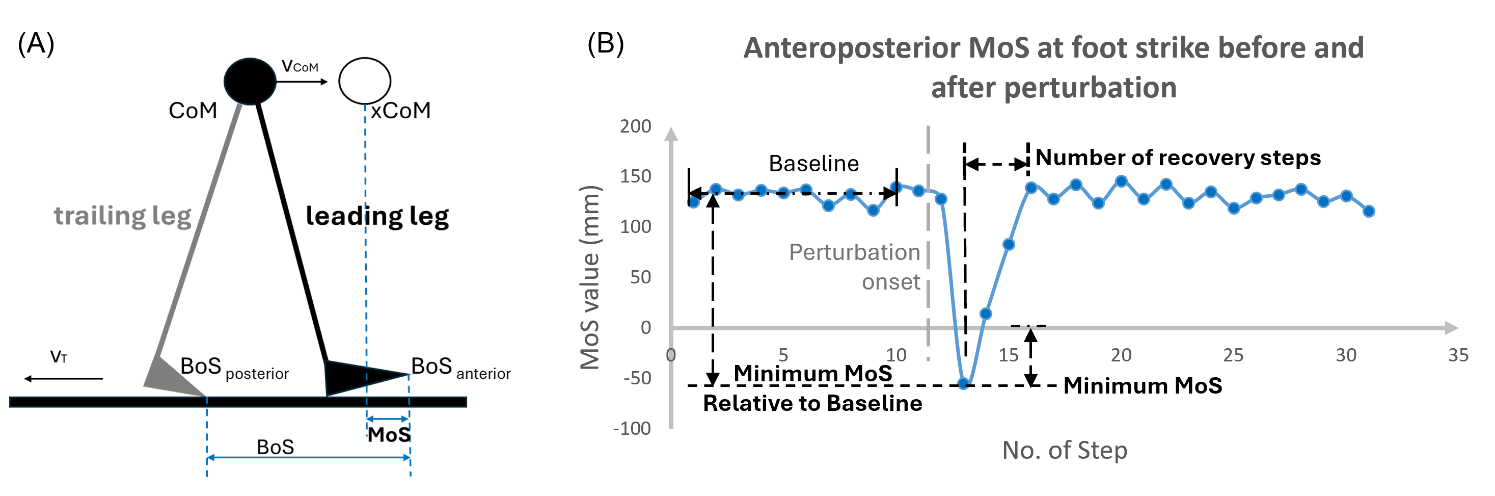


Supplementary Figure 2. Calculation and analysis of margin of stability (MoS). (A) Schematic illustration of anteroposterior MoS during treadmill walking. CoM: center-of-mass position. xCoM: extrapolated center-of-mass position. v_CoM_: center-of-mass velocity relative to ground. v_T_: treadmill belt velocity relative to ground. BoS _anterior_: toe marker position of leading leg. BoS _posterior_: toe marker position of trailing leg. (B) Illustration of analyzed parameters of anteroposterior MoS during treadmill walking.


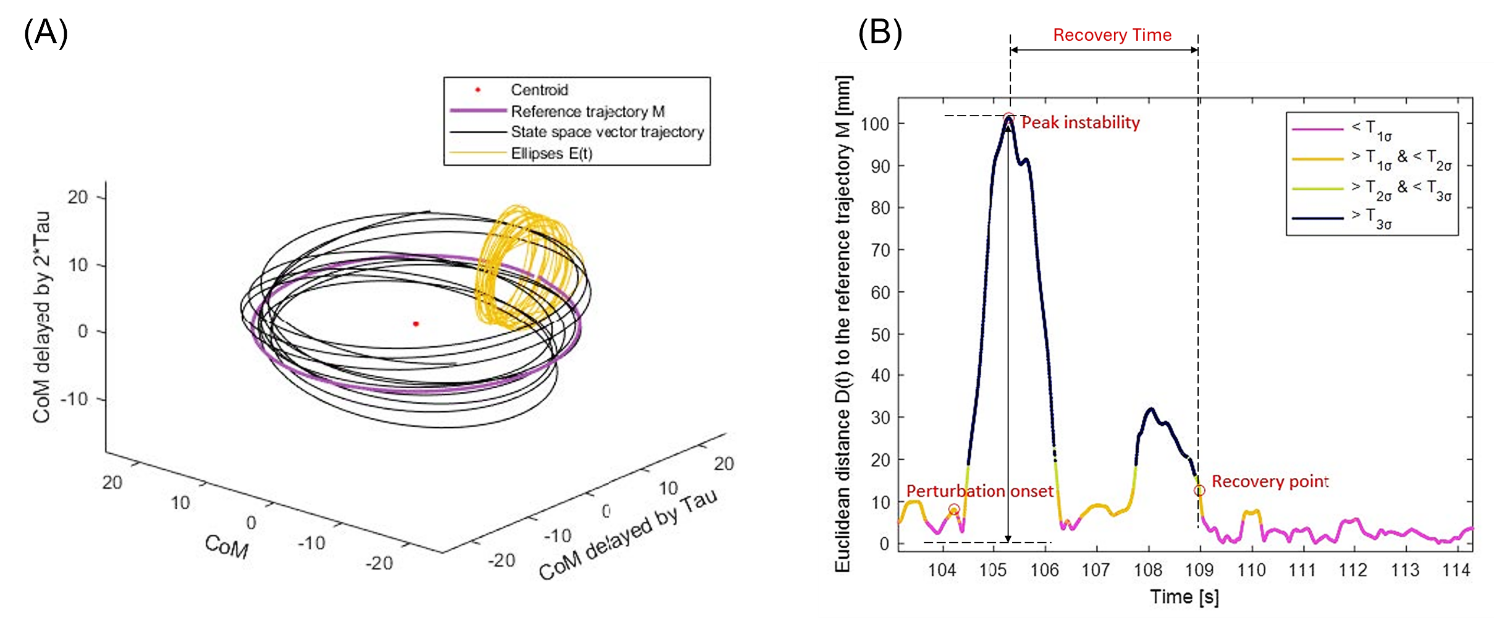


Supplementary Figure 3. Processing and analysis of resilience. (A) Illustration of using state space reconstruction of vertical center of mass (CoM) displacement time series to obtain steady state. The ellipses E around the reference trajectory M indicate the boundaries for steady-state behavior. (B) Euclidean distances of state space vectors from the reconstructed post-perturbation trajectory to the reference trajectory M.
